# Safeguarding traditional Chinese medicine intangible cultural heritage and enabling its potential public health reuse: a theory-informed qualitative study in Jiangsu, China

**DOI:** 10.3389/fpubh.2026.1886809

**Published:** 2026-09-11

**Authors:** Jie Li, Shuxin Dong, Huanhuan Liu, Junlong Shen

**Affiliations:** School of Health Economics and Management, Nanjing University of Chinese Medicine, Nanjing, China

**Keywords:** heritage governance, intangible cultural heritage, potential public health reuse, Schellenberg’s dual-value theory, theory-informed qualitative analysis, traditional Chinese medicine

## Abstract

**Background:**

TCM intangible cultural heritage (ICH) preserves medical knowledge, embodied therapeutic and pharmaceutical skills, and culturally embedded health practices. This study examined how such heritage may be safeguarded as a living knowledge system while enabling potential reuse in public health-related settings.

**Methods:**

We conducted an abductive, theory-informed qualitative analysis using grounded theory coding procedures. Fifteen semi-structured stakeholder interviews constituted the primary analytical dataset, while 32 policy documents, safeguarding reports, public heritage descriptions, and media materials were used for contextualization and triangulation. Data-led open and axial coding generated 17 categories and six main categories. Only after this category system had stabilized was Schellenberg’s dual-value theory used for post-coding interpretation.

**Results:**

The data-led stage generated 17 categories and six main categories. A subsequent theory-informed stage organized the six main categories into three higher-order interpretive domains: internal capabilities, external drivers, and amplification pathways. The model proposes that public-health-related reuse may generate resources, recognition, institutional support, and transmission incentives that feed back into primary-value safeguarding. The higher-order domains and the primary/secondary-value mapping constitute post-coding analytical interpretations.

**Conclusion:**

TCM ICH can be understood as a living cultural and knowledge-practice resource with potential public health relevance. The findings offer a provisional, mechanism-generating account of interactions among safeguarding, institutional support, communication, technology, and regulated market activity. Evaluation of causal effects and population-level health outcomes requires further research.

## 1 Introduction

Traditional medical knowledge has renewed relevance in contemporary public health because it is closely associated with disease prevention, self-care, community-based health practices, chronic disease management, and culturally embedded health beliefs (1–3). As a major component of traditional medical knowledge in China, TCM ICH preserves historically accumulated diagnostic and therapeutic skills, pharmaceutical processing techniques, health-preserving practices, and medical cultural values (4–6). These resources function as cultural symbols and may also serve as public health resources that contribute to health education, preventive care, community health services, and the cultural acceptability of health interventions (7–9).

However, the public health potential of TCM ICH cannot be realized through preservation alone (10–12). Many heritage items rely on embodied skills, tacit knowledge, family- or school-based transmission, and long-term trust between practitioners and local communities (13–15). When these resources are detached from living practice, they may become static cultural exhibits (16–18). Conversely, when utilization is driven solely by commercialization or efficacy extraction, the cultural and technical foundations of heritage may be weakened (10, 13, 17). Therefore, a key challenge is how to protect the authenticity and continuity of TCM ICH while enabling its appropriate transformation into public health resources.

Jiangsu Province provides a theoretically informative setting because its long-standing TCM traditions include regional medical schools such as Wumen, Menghe, Shanyang, and Longsha (19). These traditions encompass clinical knowledge, pharmaceutical processing, apprenticeship, institutional safeguarding, and contemporary forms of communication and utilization. The province was selected to examine relationships among living transmission, governance support, public accessibility, and potential public-health-related reuse; province-level prevalence estimation lay outside the study scope.

Existing studies have discussed the safeguarding, transmission, and productive utilization of ICH and TCM ICH from perspectives such as cultural preservation, collaborative governance, industrial development, and digital communication (20–22). However, relatively limited attention has been paid to how TCM ICH can be understood as a dual-value system that simultaneously preserves cultural and technical authenticity while generating broader public health, cultural dissemination, and socio-economic value (23–25). In particular, the mechanisms through which heritage bearers, policy support, market demand, communication innovation, and technological empowerment interact to shape public health-oriented utilization remain insufficiently clarified.

To address this gap, this study uses grounded theory coding procedures within an abductive, theory-informed qualitative design. It first develops categories from interviews and supplementary documents and then uses Schellenberg’s dual-value theory for post-coding theoretical integration. The study constructs an interpretive ‘internal capabilities–external drivers–amplification pathways’ model while explicitly distinguishing empirical category generation from theoretically informed mapping.

Accordingly, the central research question is: How can TCM ICH preserve its primary value while generating secondary value in public-health-related contexts? The analysis focuses on mechanisms through which safeguarding, transmission, communication, and utilization may create potential public health utility.

## 2 Literature review and theoretical framework

### 2.1 TCM ICH and public health resources

TCM ICH refers to medical knowledge, diagnostic and therapeutic skills, pharmaceutical processing techniques, health-preserving practices, and cultural expressions that have been transmitted across generations (16–18). Unlike static cultural objects, TCM ICH is embedded in living practice and is sustained through embodied skills, tacit knowledge, community trust, and intergenerational transmission (10–12). It therefore contains both cultural meanings and health-related functions.

From a public health perspective, TCM ICH combines cultural-preservation value with potential contributions to health education, preventive care, community-based health services, chronic disease self-management, and culturally acceptable health interventions (1–3). Many TCM ICH items embody locally accumulated experiences concerning disease prevention, seasonal health preservation, constitution-based adjustment, and the use of traditional medical techniques (4–6). These experiences may help connect formal health systems with community-level health needs, especially in contexts where culturally familiar and locally trusted resources facilitate public participation (7–9).

Transforming TCM ICH into public health resources requires a coordinated process extending beyond documentation, exhibition, or commercially driven extraction. Protection confined to documentation and exhibition may weaken connections with everyday health practice (10–12), whereas market- or technology-centered utilization may erode the cultural and epistemic foundations that sustain transmission (13–15). Effective transformation therefore depends on a balance among authenticity, accessibility, public value, and long-term continuity.

In this study, the term ‘public health resource’ denotes a knowledge-practice resource that can be mobilized to support health education, preventive care, community participation, culturally acceptable health communication, and access to locally trusted health-related practices. This analytical definition concerns potential utility in these settings. Correspondingly, ‘public health transformation’ describes the movement of TCM ICH from heritage preservation into settings such as community health education, preventive self-care, health promotion for the older population, chronic disease-related health literacy, and culturally embedded health communication.

Landscape and semiotic perspectives further clarify the public health relevance of TCM ICH communication. TCM museums, historical pharmacies, medical schools, processing workshops, community activities, and heritage exhibitions form cultural landscapes in which medical knowledge becomes visible, spatialized, and socially recognizable (19–39). Diagnostic and therapeutic skills, processing tools, prescription texts, apprenticeship rituals, and local medical narratives function as signs that convey trust, authority, regional identity, and cultural legitimacy. These symbolic and spatial dimensions explain how heritage resources may support health education and culturally acceptable communication.

### 2.2 Existing research on safeguarding and utilization of TCM ICH

Existing studies have examined the safeguarding and utilization of ICH from multiple perspectives, including cultural preservation, productive safeguarding, collaborative governance, digital communication, and industrial integration (28–30). In the field of TCM ICH, scholars have emphasized the importance of protecting heritage bearers, documenting traditional skills, improving institutional support, and promoting integration with cultural tourism, health services, and health-related industries (31–33). These studies have moved beyond a preservation-only orientation and increasingly recognize the need to activate heritage resources in contemporary social contexts (34–36).

Some studies have further pointed out that the living transmission of TCM ICH depends on the interaction of multiple actors, including governments, medical institutions, heritage bearers, enterprises, research institutions, and the public (37–39). Policy support provides institutional conditions and resource guarantees; heritage bearers preserve technical authenticity and cultural continuity; market demand creates opportunities for service-based and product-based transformation; communication and digital technologies expand public visibility and social recognition (20–22). These perspectives provide an important foundation for understanding the multi-factor mechanisms involved in TCM ICH governance.

Nevertheless, several gaps remain. First, much of the existing literature focuses on protection strategies or utilization pathways, while insufficient attention has been paid to the mechanism through which cultural value, medical value, public health value, and socio-economic value interact (23–25). Second, although heritage bearers, policy support, market demand, communication, and technology have been discussed separately, their relationships within an integrated analytical framework remain unclear. Third, the public health significance of TCM ICH has not been fully theorized (1–3). In particular, more research is needed to explain how traditional medical heritage can be transformed into accessible and sustainable public health resources while maintaining its cultural and technical foundations.

### 2.3 Schellenberg’s dual-value theory as an analytical lens

Schellenberg’s dual-value theory was employed as a heuristic framework for examining how knowledge-bearing resources move from their original contexts of production and use to broader forms of social reuse (40). Its relevance to TCM ICH rests on a shared analytical concern: the preservation of historically accumulated knowledge, embodied practices, institutional memory, and cultural meanings alongside the expansion of later social value. This use of the theory provides an interpretive lens while respecting the distinct characteristics of archival records and living medical heritage.

For TCM ICH, primary value refers to the original practice value generated within transmission communities, medical lineages, families, schools, and clinical or pharmaceutical practice settings. It includes lineage-based knowledge, technical authenticity, embodied skills, practitioner experience, medical ethics, and cultural identity. Primary value therefore supplies the technical and cultural foundation that distinguishes TCM ICH from ordinary health products, tourism symbols, and generalized cultural narratives.

Secondary value refers to the public reuse value generated when TCM ICH is recontextualized within contemporary public-health-related settings. It may encompass health education, preventive care, community-based services, cultural communication, health-related social participation, and appropriate economic support for continued transmission. The concept captures broader social usefulness arising from accessible, understandable, and culturally meaningful heritage knowledge.

**Figure 1:**
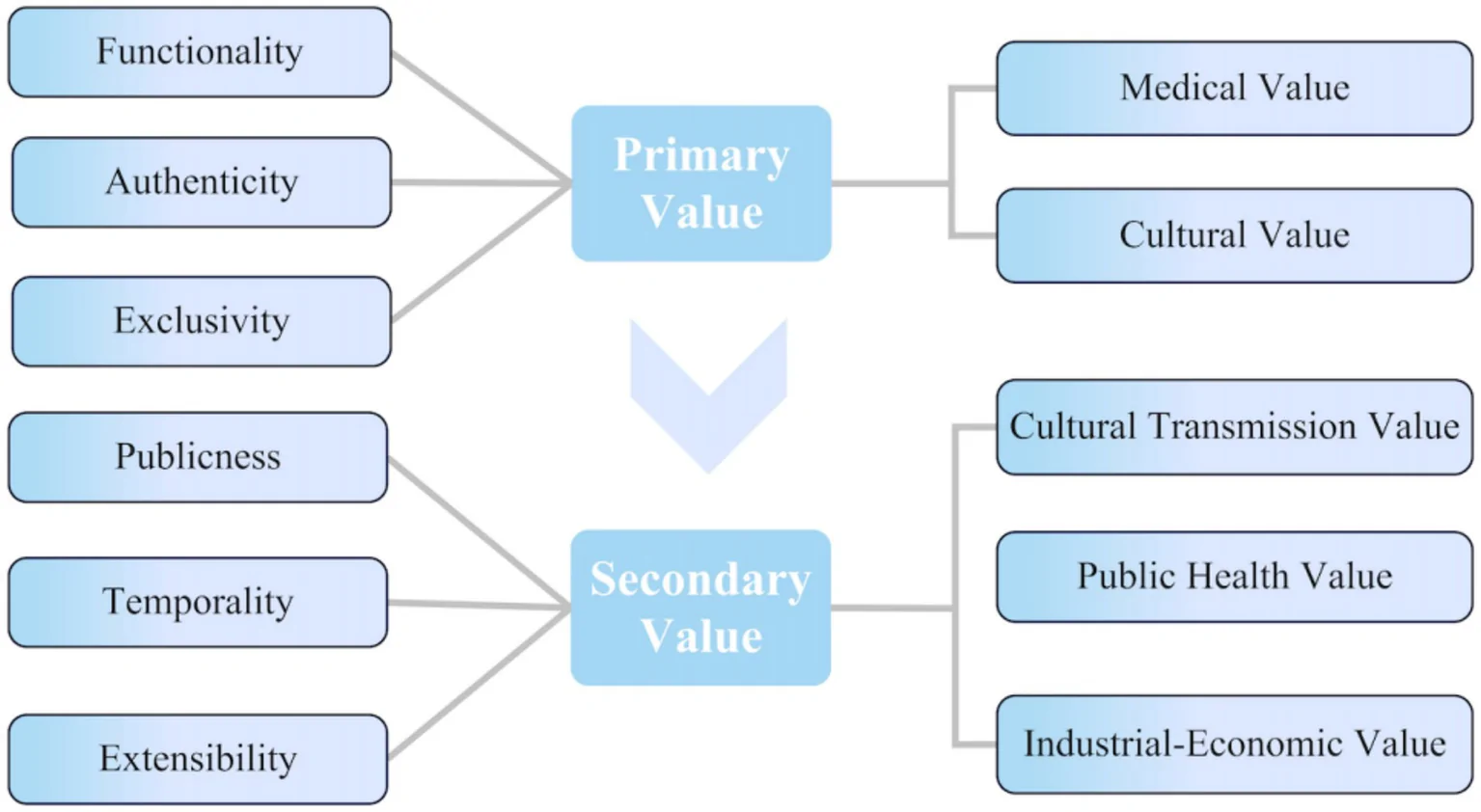
Dual values of TCM ICH based on Schellenberg’s dual-value theory.

The analytical advantage of the dual-value framework lies in its ability to connect authenticity safeguarding with public accessibility expansion (40). Cultural governance helps explain multi-actor coordination, knowledge translation clarifies how knowledge enters practice, and health communication explains information dissemination and audience engagement. The central analytical issue is how TCM ICH can protect its original cultural and technical foundation while expanding into public-health-related contexts. Dual-value theory provides an integrative lens for examining this relationship. The dual-value structure of TCM ICH based on Schellenberg’s theory is shown in Figure 1.

### 2.4 Research gap and analytical framework

Based on the above discussion, this study examines TCM ICH as a living knowledge system with two interconnected value dimensions. Heritage bearers, embodied skills, medical lineages, and cultural identity sustain primary value, whereas public-health-oriented services, education, cultural communication, market application, and digital dissemination may extend secondary value. Existing research has not fully explained how safeguarding and public reuse interact within a single analytical framework.

Accordingly, the study adopts an abductive, theory-informed qualitative strategy using grounded theory coding procedures (40–44). Schellenberg’s dual-value theory informed the research question and sensitized the researchers to the tension between safeguarding and public reuse. The initial codebook remained data-led and excluded primary value and secondary value as predefined nodes, thereby preserving an analytical distinction between empirical category generation and subsequent theoretical interpretation.

## 3 Materials and methods

### 3.1 Study setting

Jiangsu Province was selected as the empirical setting because its diverse TCM traditions and institutional actors provide a suitable case for examining living transmission, cultural identity, policy support, market activity, communication, and technological adaptation. The public registry maintained by the Jiangsu Provincial Intangible Cultural Heritage Protection Center contained 342 active Traditional Medicine ICH item records in the October 2025 snapshot used for this study (45). These records provide provincial context and remained analytically separate from the 15-person interview sample and the grounded-theory coding dataset.

**Figure 2:**
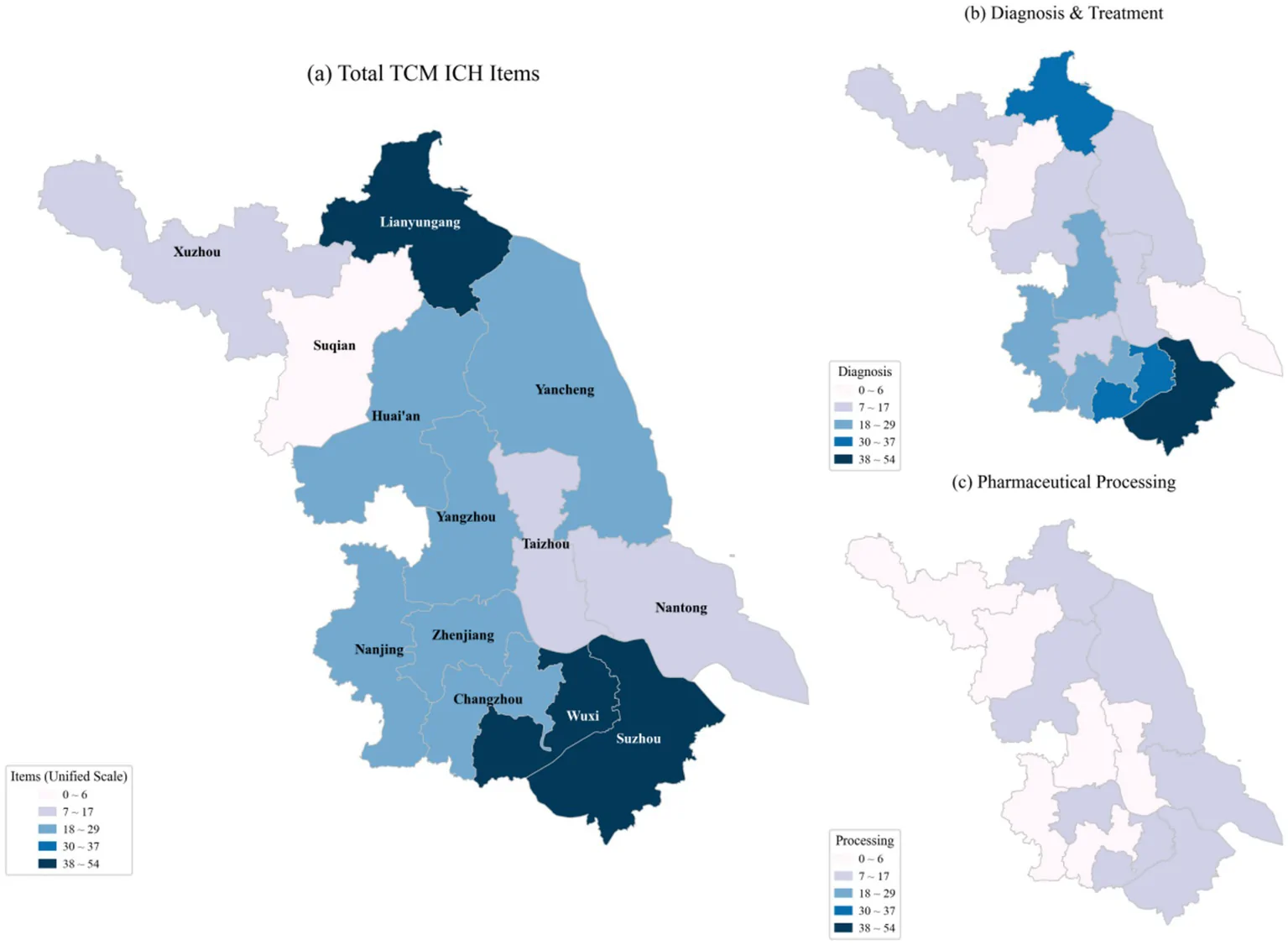
Spatial distribution of TCM ICH items in Jiangsu Province. Panels show **(a)** total listed items, **(b)** diagnosis and treatment items, and **(c)** pharmaceutical processing items by city. Source: Authors’ aggregation of the Jiangsu Provincial Intangible Cultural Heritage Protection Center public registry, Traditional Medicine (IX) category, October 2025 snapshot (45). All included heritage-item records were assigned to the city indicated in the registry.

**Figure 3:**
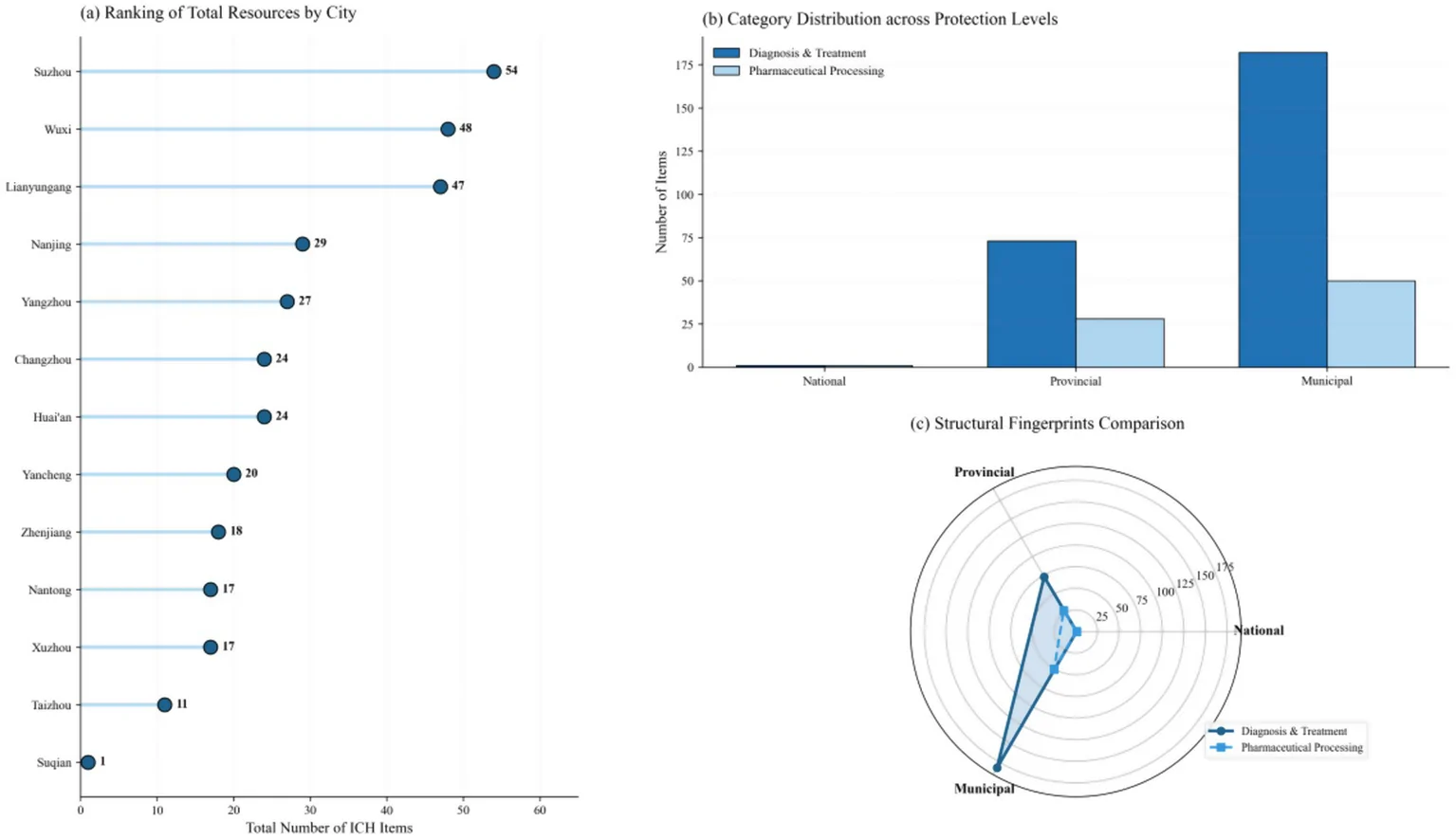
Composition of TCM ICH items in Jiangsu Province. Panels show **(a)** city ranking of item records, **(b)** practice-type counts across protection levels, and **(c)** structural comparison. Source: Authors’ aggregation of the Jiangsu Provincial Intangible Cultural Heritage Protection Center public registry, Traditional Medicine (IX) category, October 2025 snapshot (45).

**Figure 4:**
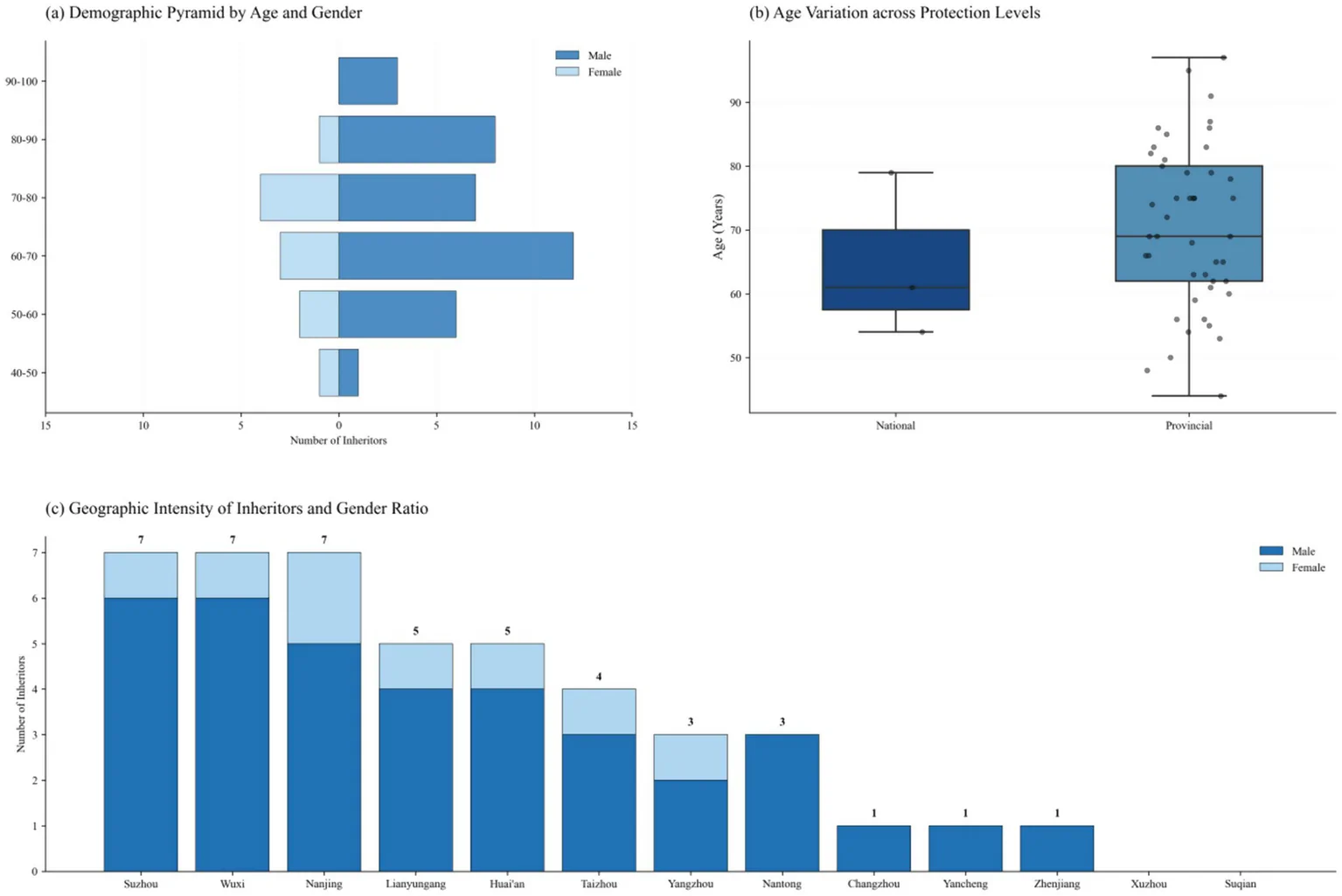
Demographic characteristics of nationally and provincially recognized TCM ICH bearers in Jiangsu Province by age, sex, protection level, and city. Source: Authors’ aggregation of the Jiangsu Provincial Intangible Cultural Heritage Protection Center public registry, Traditional Medicine (IX) category, October 2025 snapshot (45). Counts in each panel reflect bearer records for which the corresponding attribute is reported in the registry.

Figures 2–4 summarize a separate October 2025 snapshot of the public registry maintained by the Jiangsu Provincial Intangible Cultural Heritage Protection Center. Records were filtered to the Traditional Medicine (IX) category and active status, and repeated identifiers were counted once. Figures 2, 3 aggregate heritage-item records by the city, protection level, and practice type reported in the registry; all included heritage items have an explicit city attribution. Figure 4 summarizes nationally and provincially recognized representative bearers by age, sex, protection level, and city. These registry data provide descriptive provincial context and were not used in the grounded-theory coding (45).

### 3.2 Research design

This study adopted an abductive, theory-informed qualitative design using grounded theory coding procedures (41–44). The design combined data-led category development with a subsequent stage of theoretically informed interpretation. The dual-value framework shaped the research question and broad interview topics, while the coding framework was developed from the empirical material.

Analysis proceeded in two distinct stages. In Stage 1, two researchers conducted line-by-line open coding, constant comparison, memo writing, and axial integration using participant-centered expressions and empirically derived relationships. This stage generated 17 categories and six main categories. Stage 2 began after stabilization of the six-category structure and used abductive comparison with Schellenberg’s theory to organize the empirical categories into three higher-order interpretive domains and examine possible primary-to-secondary-value relationships. The study therefore reports the 17 categories and six main categories as empirically generated and the three domains and dual-value mapping as a theory-informed synthesis.

### 3.3 Participants and sampling

Maximum variation sampling recruited participants with diverse experience in the protection, transmission, research, management, and utilization of TCM ICH (46, 47). Sampling prioritized theoretical richness across stakeholder positions (46, 47). The final sample included 15 stakeholders from five groups: six heritage bearers, four experts and scholars, two enterprise managers, one government official, and two veteran pharmacy craftsmen. These participants represented different administrative levels, heritage categories, professional backgrounds, and regional contexts within Jiangsu Province. Table 1 summarizes their characteristics.

**Table 1:** Basic information of participants.

Type	Number	Representative items/research areas	Geographic distribution	Notes
ICH bearers	6	Ding’s Hemorrhoid Medical Techniques; Zhihétang paste-formula making techniques; Wumen school paste-formula making techniques; Menghe medical school, etc.	Nanjing; Wuxi; Suzhou; Nantong	National-level bearers: 2; provincial-level: 3; municipal-level: 1
Experts and scholars	4	TCM culture research; TCM literature and academic-school studies; policy research on safeguarding and utilization of TCM ICH, etc.	Nanjing; Yangzhou; Lianyungang	University-based TCM culture researchers: 2; institute of TCM schools: 1; TCM museum: 1
Enterprise managers	2	Development of ICH-derived products; industrial-chain operations	Changzhou; Lianyungang	Heads of industrialization-oriented ICH enterprises
Government official	1	Policy formulation and implementation for ICH safeguarding	Nanjing	ICH Division, Provincial Department of Culture and Tourism
Veteran pharmacy craftsmen	2	Experts for the Jiangsu Veteran Pharmacy Craftsmen Studio Project	Wuxi; Lianyungang	Veteran craftsmen with over 40 years of experience in Chinese materia medica processing: 2

The heritage bearers were associated with representative items such as Ding’s Hemorrhoid Medical Techniques, Zhihétang paste-formula making techniques, Wumen school paste-formula making techniques, and the Menghe medical school. The experts and scholars were mainly engaged in TCM culture, TCM literature, academic-school studies, and policy research on the protection and utilization of TCM ICH. The enterprise managers provided perspectives on product development and industrial-chain operations, while the government official offered insights into policy formulation and implementation. The veteran pharmacy craftsmen contributed long-term practical experience in Chinese materia medica processing.

Participants were recruited through purposive and maximum variation sampling. The research team first identified potential participants through provincial and municipal TCM ICH lists, academic networks, TCM cultural institutions, and enterprise or project contacts, and then used targeted snowball recommendations to locate stakeholders with direct experience of safeguarding, transmission, management, research, or utilization. Inclusion criteria were: adult age, at least 3 years of relevant experience with TCM ICH or TCM cultural work, willingness to participate in an audio-recorded interview, and ability to provide information about at least one aspect of protection, transmission, public communication, policy support, or utilization. Exclusion criteria were: no direct involvement with TCM ICH, inability to complete an interview, or unwillingness to provide informed consent.

The sample was designed to maximize theoretical richness. Recruitment continued until the emerging categories incorporated the major stakeholder perspectives and additional interviews ceased to generate new categories. Purposive recruitment through professional networks precluded calculation of a formal refusal rate. The limited number of government and enterprise participants positions policy- and market-related findings as exploratory and mechanism-generating. Inclusion of participants from southern, central, and northern Jiangsu broadened regional coverage, while transferability to other Chinese regions requires comparative research.

### 3.4 Data collection

Data were collected mainly through semi-structured interviews (48, 49). The interview guide addressed safeguarding, transmission difficulties, institutional support, public awareness, market activity, technological application, and the broad tension between cultural continuity and public reuse. Neutral topic prompts allowed participants to frame these issues in their own terms; primary value, secondary value, internal capabilities, external drivers, and amplification pathways were introduced only during post-coding interpretation.

Each interview lasted approximately 40–60 min and was audio-recorded with the participant’s consent. After the interviews, the recordings were transcribed verbatim and anonymized before analysis. Field notes were used to supplement the interview transcripts, especially when participants referred to local practices, specific heritage items, or contextual details that required further clarification.

To enhance credibility, the study collected 32 supplementary materials, including policy documents, safeguarding work reports, publicly available heritage descriptions, and media coverage. The 15 interview transcripts constituted the primary dataset for category development. Documentary materials supported contextualization, cross-source comparison, and triangulation of factual claims. The administrative-registry snapshot used for Figures 2–4 formed a third contextual source. Each source therefore served a distinct analytical function: interviews supported category generation, documents supported contextual interpretation and triangulation, and registry records supported provincial description.

### 3.5 Data analysis

NVivo Plus 11 supported qualitative data management. Stage 1 comprised line-by-line open coding, constant comparison, memo writing, and axial coding (41–44). Two researchers retained *in vivo* or near-participant expressions where possible and compared statements within and across stakeholder groups. Code nodes and category labels at this stage were grounded in the empirical material; dual-value terminology entered the analysis during Stage 2.

Constant comparison was conducted within and across participant groups and against the supplementary documents. Concepts were merged only when they referred to the same condition, action, trade-off, or consequence. Memos recorded decisions to retain, merge, split, or elevate codes and documented divergent cases, including circumstances in which market activity or technological adoption supported transmission as well as circumstances in which they weakened authenticity or bearer authority. Stage 1 produced 17 categories and six main categories.

During axial coding, the 17 categories were integrated into six main categories according to their empirical relationships in the safeguarding-utilization process: policy support and institutional safeguards; bearer-related factors; communication and awareness raising; innovation and technological integration; transmission and identification of cultural values; and market expansion and industrial development. Stage 2 commenced after stabilization of this structure. Abductive interpretation then organized the six categories as internal capabilities, external drivers, and amplification pathways and mapped them onto Schellenberg’s dual-value distinction. This mapping is designated throughout the manuscript as a post-coding analytical synthesis.

### 3.6 Coding reliability and theoretical saturation

To improve analytical reliability, two researchers independently coded the interview transcripts and compared code names, category boundaries, and supporting excerpts (48, 49). Discrepancies were resolved through discussion, and a third researcher reviewed the six-category structure and selected evidence. The audit trail records separate outputs for the data-led coding stage and the theory-informed integration stage, allowing readers to distinguish empirical categories from post-coding interpretation.

Analytical sufficiency was assessed by distinguishing code saturation from meaning saturation (50, 51). After stabilization of the initial category system, three additional interviews were coded using the Stage 1 procedures. They generated no new categories and deepened existing categories, particularly market expansion and technological integration. These results support code saturation for the six-category empirical framework while leaving open further development of meaning and alternative theoretical mappings.

## 4 Results

### 4.1 Open coding

Open coding identified concepts and categories from the interview materials through a coding framework independent of the dual-value terminology. Statements concerning safeguarding, transmission, utilization, accessibility, and health-related social use were coded close to participants’ wording and compared across cases. This process generated 17 categories covering institutional, human, communicative, technological, cultural, and market-related conditions.

Open coding associated safeguarding and potential public reuse with institutional, human, cultural, technological, communicative, and market-related conditions. Participants emphasized resource identification, bearer cultivation, sustained support, public communication, and context-sensitive adaptation. These findings identify qualitative relationships among the conditions shaping safeguarding and public reuse.

To increase transparency, Table 2 shows how selected interview or documentary excerpts were condensed into initial concepts and connected to the 17 categories. Table 3 presents the data-led integration of these categories into six main categories. Table 4 reports the subsequent theoretical mapping, and Table 5 specifies the analytical boundary between empirical category generation and theory-informed interpretation.

**Table 2:** Examples of data-led open coding using interview and documentary excerpts.

Main category	Initial concept	Category	Illustrative evidence excerpt (interview or document)	Data-led analytical interpretation
A Policy support and institutional safeguards	Resource-base clarification and funding guarantee	A1 Inventory survey; A4 Financial and resource inputs	“If we do not know our resource base clearly, comprehensive protection is impossible.” “Strong funding is a solid guarantee for the inheritance and development of ICH.”	These statements show that public health-oriented transformation first depends on knowing what resources exist and whether continuous institutional and financial inputs are available.
B Bearer-related factors	Successor shortage and long-term discipline	B1 Training and capacity building; B2 Number of bearers	“The shortage of successors is one of the major difficulties facing ICH transmission.” “Bearers need to settle down, be patient, and devote 10 years to sharpening one skill.”	The quotation links primary value to embodied skills, apprenticeship, and the time needed to maintain technical authenticity.
C Communication and awareness raising	Public visibility and educational outreach	C1 Public education; C2 Communication channels	“TCM ICH should not be kept in seclusion and unknown to the public.” “ICH was brought into campuses, communities, and classrooms.”	Communication is not only publicity; it is a pathway through which heritage knowledge becomes accessible for health education and community participation.
D Innovation and technological integration	Living wisdom and adaptive innovation	D1 Updating the paradigm; D2 Integration of modern technologies	“ICH is not a specimen placed in a museum, but living wisdom that can solve contemporary problems.” “Tradition and innovation are not contradictory; they coexist.”	Innovation is interpreted as adaptive continuation rather than replacement of tradition, especially when technology supports recording, interpretation, and safer contemporary use.
E Transmission and identification of cultural values	Ethical value and cultural identity	E1 Transmission of values; E2 Cultural identity	“There is no end to learning pharmacy, and there is no trivial matter in pharmacy.” “The essence of ICH protection is not only to protect techniques, but first to protect ideas and values.”	These materials indicate that medical ethics, responsibility, and cultural meaning are part of primary value rather than external decoration.
F Market expansion and industrial development	Return to everyday life and economic balance	F1 Market demand; F3 Industrial-chain development; F4 Economic benefits	“Processing techniques that had become detached from everyday life should return to contemporary life and serve people’s health.” “A dynamic balance between machinery and handcraft, cost and price, is a key challenge.”	Market transformation can support secondary value only when it remains connected to health-related daily life and does not erode the technical and cultural basis of heritage.

**Table 3:** Results of axial coding.

Main category	Category	Core meaning	Logic of category linkage
A Policy support and institutional safeguards	A1 Inventory survey of TCM cultural resources	Provide a systematic regulatory and institutional framework for TCM ICH, balancing cultural safeguarding and medical norms, and ensuring conditions in people, finance, materials, and information for safeguarding and utilization.	Resource inventory (A1) provides a basis for protection-level designation (A2); institutional design (A3) safeguards the precision of resource inputs (A4).
	A2 Designation of protection levels		
	A3 Institutional design and legal safeguards		
	A4 Financial and resource inputs		
B Bearer-related factors	B1 Training and capacity building of bearers	Bearer-related factors constitute the core carrier linking traditional skills and modern society and are key to ensuring smooth intergenerational transmission.	Bearer training systems (B1) influence numerical and structural profiles (B2), while economic benefits (F4) feed back into willingness to transmit (B3).
	B2 Number of bearers		
	B3 Willingness to transmit		
C Communication and awareness raising	C1 Public education and science popularization outreach	Expand dissemination coverage, visibility, and influence of TCM ICH through multiple channels; foster a favorable social atmosphere; and consolidate the social foundation for safeguarding and utilization.	Science popularization (C1) should be combined with innovative means such as VR (C2) to strengthen cultural identity (E2).
	C2 Diversity of communication methods and channels		
D Innovation and technological integration	D1 Updating the safeguarding-and-utilization paradigm	Update concepts and empower traditional skills with technology, balancing authenticity and modern adaptability to better meet contemporary needs and improve the effectiveness of safeguarding and utilization.	Paradigm updating (D1) guides the direction of technology application (D2); for example, quantitative research on clinical effectiveness increases market recognition (F1).
	D2 Integration of modern technologies with traditional practices		
E Transmission and identification of cultural values	E1 Transmission of values	Transmit core TCM thinking so that the public develops dual identification with the cultural and medical value of TCM ICH, providing cultural foundations and spiritual motivation for safeguarding and utilization.	Transmission of core values (E1) is the basis of cultural identity (E2), while clinical recognition (E2) becomes a driver of value transmission.
	E2 Cultural identity		
F Market expansion and industrial development	F1 Market demand	Promote deep integration between TCM ICH and industry to realize its economic value and feed back into safeguarding and transmission.	Market demand (F1) guides industrial-chain improvement (F3), while economic benefits (F4) enhance market expansion capacity (F2).

**Table 4:** Post-coding theoretical mapping of empirically generated categories.

Empirically generated main category	Constituent categories	Post-coding interpretive domain	Theory-informed relation to dual value
Policy support and institutional safeguards	A1 Resource inventory; A2 Protection-level designation; A3 Institutional design; A4 Resource inputs	External drivers	Primarily enables secondary-value reuse while also providing conditions for primary-value safeguarding.
Bearer-related factors	B1 Training; B2 Number of bearers; B3 Willingness to transmit	Internal capabilities	Interpreted as a principal carrier of primary value and intergenerational continuity.
Communication and awareness raising	C1 Public education; C2 Communication channels	Amplification pathways	May expand secondary-value reach and return recognition to primary-value safeguarding.
Innovation and technological integration	D1 Paradigm updating; D2 Technology integration	Amplification pathways	May document primary value and expand accessibility, but may also create substitution risks.
Transmission and identification of cultural values	E1 Value transmission; E2 Cultural identity	Internal capabilities	Interpreted as the cultural and ethical content of primary value.
Market expansion and industrial development	F1 Market demand; F2 Expansion capacity; F3 Industrial chain; F4 Economic benefits	External drivers	May support secondary value and feed resources back to primary value; effects are conditional on governance.

**Table 5:** Distinction between empirically generated categories and theory-informed interpretation.

Analytical stage	Procedure and evidence base	Output in this study	Analytical status
Open coding (Stage 1)	Line-by-line coding of interview transcripts using *in vivo* or near-participant expressions.	Initial concepts (e.g., bearer shortages, limited communication channels, and short industrial chains) and 17 categories.	Empirically generated
Constant comparison and axial integration (Stage 1)	Comparison within and across stakeholder groups, followed by integration through observed relationships in the safeguarding-utilization process.	Six main categories: institutional safeguards; bearer-related factors; communication; technology; cultural-value transmission; and market development.	Empirically generated
Category-structure stabilization (Stage 1)	Memo writing, category-boundary review, and negative-case analysis of enabling and potentially erosive mechanisms.	Refined category boundaries, relational structure, and empirically ambivalent market and technology cases.	Empirically grounded and analytically audited
Post-coding theoretical integration (Stage 2)	Abductive comparison with Schellenberg’s theory after stabilization of the six-category framework.	Internal capabilities, external drivers, and amplification pathways.	Theory-informed interpretation
Dual-value mapping (Stage 2)	Interpretive mapping of empirical categories to primary-value safeguarding and potential secondary-value reuse.	Primary-value foundation and secondary-value application domains.	Theory-informed interpretation
Dynamic feedback proposition (Stage 2)	Synthesis of evidence on funding, recognition, institutional support, transmission incentives, and governance risks.	Provisional feedback loop and governance guardrails.	Provisional theory-informed proposition
Analytical sufficiency	Three additional interviews coded using Stage 1 procedures; no new categories were generated.	Code saturation for the six-category empirical framework.	Applies to the empirical framework; alternative theoretical mappings remain open

### 4.2 Axial coding

Axial coding examined relationships among the 17 categories generated in Stage 1. Categories with related conditions, actions, trade-offs, or consequences were integrated into six empirical main categories: policy support and institutional safeguards; bearer-related factors; communication and awareness raising; innovation and technological integration; transmission and identification of cultural values; and market expansion and industrial development. The results are summarized in Table 3.

The first main category, policy support and institutional safeguards, reflects the institutional conditions required for identifying, classifying, funding, and regulating TCM ICH. Resource inventory provides the basis for protection-level designation, while institutional design and financial input determine whether protection measures can be implemented continuously. The second main category, bearer-related factors, concerns the human carriers of heritage knowledge. Training mechanisms, the number of bearers, and willingness to transmit directly influence the continuity of embodied skills and tacit knowledge.

The third main category, communication and awareness raising, refers to the expansion of public visibility and social recognition through health education, science popularization, media communication, exhibitions, and experiential activities. The fourth main category, innovation and technological integration, reflects the role of modern technologies in recording, interpreting, disseminating, and adapting traditional medical knowledge. The fifth main category, transmission and identification of cultural values, emphasizes the importance of TCM thinking, medical ethics, regional identity, and public trust. The sixth main category, market expansion and industrial development, concerns the transformation of heritage resources into services, products, and health-related industries under appropriate governance conditions.

For policy support and institutional safeguards, the interview and documentary materials emphasized that resource identification is a prerequisite for protection. One excerpt stated, ‘If we do not know our resource base clearly, comprehensive protection is impossible.’ This statement was coded as baseline inventory and then integrated into the category of resource inventory because it shows that public-health-oriented transformation depends on identifying, classifying, and institutionally recognizing heritage resources.

For bearer-related factors, the materials highlighted the fragility of embodied transmission. A representative excerpt noted, ‘The shortage of successors is one of the major difficulties facing ICH transmission,’ while another stressed that bearers need patience and long-term discipline. These statements support the category of bearer training and number of bearers, indicating that primary value depends on apprenticeship, practical experience, and the willingness to preserve tacit knowledge over time.

For communication and awareness raising, participants and documents described public visibility as a condition for social use. One excerpt stated that TCM ICH ‘should not be kept in seclusion and unknown to the public,’ and another referred to bringing ICH into campuses, communities, and classrooms. These excerpts explain why communication was interpreted as a public health-related pathway: it allows heritage knowledge to become understandable, accessible, and usable in health education contexts.

For innovation and technological integration, the materials did not frame innovation as a replacement for tradition. Instead, one excerpt described ICH as ‘living wisdom that can solve contemporary problems,’ and another stated that ‘tradition and innovation are not contradictory; they coexist.’ These statements support the category of paradigm updating and technological integration, suggesting that modern technology should help record, interpret, and adapt heritage practices while preserving their original knowledge base.

For transmission and identification of cultural values, the materials showed that heritage value includes ethics and identity as well as techniques. The phrase ‘there is no trivial matter in pharmacy’ was coded as professional responsibility and then integrated into value transmission. Another excerpt emphasized that ICH protection is not only the protection of techniques but also the protection of ideas and values. These data explain why cultural value was treated as part of primary value.

For market expansion and industrial development, the materials indicate that market activity may reconnect heritage with everyday health-related life and generate resources for transmission, while also creating pressures toward simplification, standardization, and detachment from cultural and technical foundations. The resulting category therefore retains this empirical ambivalence and foregrounds the governance conditions that shape market-related transformation.

### 4.3 Post-coding theoretical integration

After stabilization of the six empirical main categories, a separate theory-informed stage examined their relationship to the research question and Schellenberg’s dual-value distinction. Abductive comparison organized the six categories into three higher-order interpretive domains: internal capabilities, external drivers, and amplification pathways. These domains function as analytical constructs that synthesize the empirical categories. Table 5 records the epistemic status of each analytical stage.

The internal-capabilities domain combines bearer-related factors with the transmission and identification of cultural values. These categories were generated in Stage 1; only in Stage 2 were they interpreted in relation to primary-value safeguarding. Thus, ‘internal capabilities’ and ‘primary value’ are analytical labels introduced after coding rather than terms imposed on participants’ accounts.

The external-drivers domain combines policy support and institutional safeguards with market expansion and industrial development. Policy support can provide legitimacy, resources, and regulatory boundaries, while market activity may create opportunities for service, product, or scenario-based reuse. The analysis also retains a negative case: commercialization can erode primary value when it displaces technical standards, bearer authority, or cultural meaning.

The amplification-pathways domain combines communication and awareness raising with innovation and technological integration. These categories may expand visibility, documentation, interpretation, and accessibility, although their effects depend on context and governance. Technology may assist documentation and dissemination but may weaken hands-on apprenticeship when treated as a substitute for embodied practice. The domain therefore represents a conditional amplification mechanism.

### 4.4 Interpretive model of influencing relationships

The model advances a dynamic feedback proposition. Potential secondary-value outcomes may return financial resources, social recognition, institutional support, and transmission incentives to bearer cultivation and cultural continuity. Qualitative evidence concerning funding, recognition, market returns, and willingness to transmit supports the feedback loop as a provisional, theory-informed proposition.

**Figure 5:**
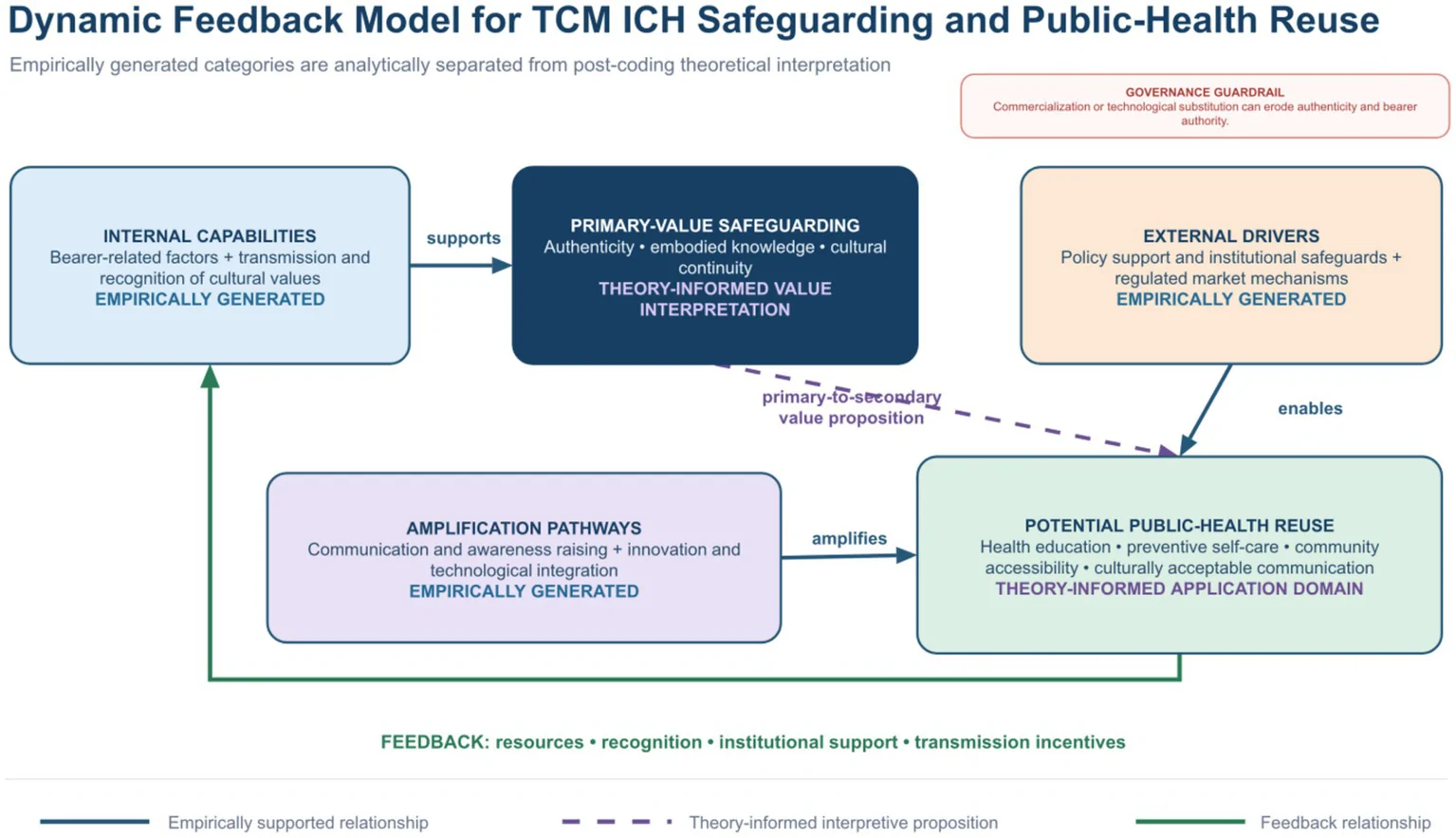
Dynamic, theory-informed feedback model for TCM ICH safeguarding and potential public-health-related reuse. Solid blue arrows denote relationships supported by interview and documentary evidence; the dashed purple arrow denotes a post-coding interpretive proposition. The green feedback path proposes that resources, recognition, institutional support, and transmission incentives may reinforce internal capabilities. Node labels distinguish empirically generated categories from theory-informed constructs.

The model also includes governance guardrails. Market expansion, communication, and technological adoption may support accessibility and continued transmission, but excessive commercialization, decontextualized communication, or technological substitution may weaken authenticity and bearer authority. Solid arrows in Figure 5 denote relationships supported by interview and documentary evidence; dashed arrows denote theory-informed interpretive propositions. The model should therefore be read as a provisional mechanism-generating framework.

In operational terms, ‘public-health-related reuse’ refers to potential utility in health education, preventive self-care, community accessibility, culturally acceptable communication, and health-equity-oriented outreach. These analytical application domains specify plausible settings for reuse, while outcome evaluation remains a task for future research. Table 6 summarizes their operational meaning.

**Table 6:** Operational meaning of public health transformation of TCM ICH.

Dimension	Operational meaning	Empirical manifestation
Health education	Using TCM ICH knowledge, stories, demonstrations, and exhibitions to improve public understanding of health-related traditional medical knowledge.	Campus, community, museum, and lecture-based activities that make traditional medical knowledge understandable to lay audiences.
Preventive care	Mobilizing health-preserving practices and locally familiar self-care knowledge in non-therapeutic or complementary preventive contexts.	Seasonal health preservation, constitution-related self-care education, and daily-life health literacy activities.
Community accessibility	Embedding heritage knowledge in community-facing scenarios so that residents can encounter and understand locally trusted health-related practices.	ICH entering communities, classrooms, pharmacies, museums, workshops, and local health promotion settings.
Cultural acceptability	Strengthening trust, identity, and cultural legitimacy in health communication through recognizable local medical traditions and symbols.	Medical-school narratives, pharmacy heritage, bearer stories, processing tools, and ritualized transmission practices.
Health equity potential	Creating possible channels through which culturally familiar and low-threshold health knowledge may reach older adults, rural residents, and groups with limited access to formal health education.	Public welfare lectures, community outreach, school-based dissemination, and locally adapted communication channels.

## 5 Discussion

### 5.1 TCM ICH as a public health resource

This study found that the protection and utilization of TCM ICH are shaped by interactions among internal capabilities, external drivers, and amplification pathways. The findings extend understanding of TCM ICH from a cultural preservation object to a living knowledge system with potential public health value (1, 2). Its diagnostic and therapeutic skills, pharmaceutical processing techniques, health-preserving practices, and medical cultural values may contribute to health education, preventive care, community-based health services, and culturally acceptable health interventions (52–54).

The public health relevance of TCM ICH lies in its capacity to connect traditional medical knowledge with contemporary health needs. Many heritage items are rooted in local practice and long-term community trust, preserving knowledge about disease prevention, seasonal health preservation, constitution-based adjustment, and daily self-care. Public health interventions draw on professional medical services as well as public understanding, cultural acceptance, and community participation (55–57), making these heritage characteristics analytically salient.

### 5.2 Dual-value coordination as an interpretive proposition

The data-led categories indicate an interdependent and potentially tension-filled relationship between safeguarding and public reuse. The subsequent dual-value interpretation identifies embodied knowledge, cultural identity, and bearer continuity as the primary-value foundation, with public communication, service scenarios, and regulated economic activity offering possible routes for extending secondary value. The present study advances this relationship as a theory-informed proposition for future empirical testing (40).

The model proposes a feedback loop: when public reuse generates resources, institutional recognition, public trust, or stronger incentives for transmission, these benefits may support heritage bearers and safeguarding. This positive effect depends on governance, because commercialization or inappropriate technology use may also weaken authenticity. The model therefore identifies both the conditions that enable reuse and the safeguards needed to protect primary value.

### 5.3 Governance implications for public health transformation

These findings have implications for heritage governance and public health governance. First, formal designation should be combined with sustained support for bearer cultivation, skill documentation, public-health-oriented application, and community dissemination (19, 26, 27). Second, communication and digital technologies should complement professional judgment, embodied skills, and bearer-led practice (39, 55, 56). Digital recording, online dissemination, and intelligent tools can improve accessibility and visibility when embedded within these professional and cultural safeguards (58–60).

Public health-oriented utilization should also be context-sensitive (28–30). Regions with strong heritage foundations and mature institutional conditions may explore community health services, health education programs, and heritage-based wellness services. Regions with weaker foundations should prioritize documentation, bearer support, and basic transmission capacity before pursuing large-scale industrial transformation. Through multi-actor collaboration among governments, heritage bearers, medical institutions, universities, enterprises, communities, and media platforms, TCM ICH can be transformed into public health resources while maintaining its cultural and technical foundations (11, 12, 37).

### 5.4 Limitations and future research

This study has three principal limitations. First, the qualitative sample comprised 15 participants from Jiangsu, including one government official and two enterprise managers. The findings therefore reflect a single-province case, and the policy- and market-related categories require examination in broader and more balanced samples. Second, Figures 2–4 are based on an October 2025 snapshot of a continuously updated public registry. They describe the provincial distribution of listed TCM ICH resources at that time and should not be interpreted as evidence of population prevalence or causal relationships. Third, the 17 categories and six main categories were generated from the qualitative data, whereas the three higher-order domains, dual-value mapping, and feedback loop are theory-informed interpretations. These propositions require further testing through multi-region, longitudinal, or mixed-method studies, particularly before conclusions are drawn about public health outcomes.

## 6 Conclusion

This study used data-led coding to generate 17 categories and six main categories concerning the safeguarding and potential public-health-related reuse of TCM ICH in Jiangsu. A subsequent theory-informed stage organized these empirical categories into internal capabilities, external drivers, and amplification pathways and mapped them onto Schellenberg’s dual-value distinction. The resulting model proposes that appropriately governed public reuse may return resources, recognition, institutional support, and transmission incentives to primary-value safeguarding. It also identifies governance risks associated with commercialization, oversimplified communication, and technological substitution. The framework offers a basis for comparative research and context-sensitive heritage governance.

## Data Availability

The raw data supporting the conclusions of this article will be made available by the authors, without undue reservation.
